# Tomato Defenses Under Stress: The Impact of Salinity on Direct Defenses Against Insect Herbivores

**DOI:** 10.1111/pce.15353

**Published:** 2025-01-13

**Authors:** Sahil V. Pawar, Sujay M. Paranjape, Grace K. Kalowsky, Michelle Peiffer, Nate McCartney, Jared G. Ali, Gary W. Felton

**Affiliations:** ^1^ Department of Entomology The Pennsylvania State University University Park Pennsylvania USA

**Keywords:** abiotic stress, biotic stress, insect herbivory, ionic toxicity, salt stress

## Abstract

Abiotic stressors, such as salt stress, can reduce crop productivity, and when combined with biotic pressures, such as insect herbivory, can exacerbate yield losses. However, salinity‐induced changes to plant quality and defenses can in turn affect insect herbivores feeding on plants. This study investigates how salinity stress in tomato plants (*Solanum Lycopersicum* cv. Better Boy) impacts the behavior and performance of a devastating insect pest, the tomato fruitworm caterpillar (*Helicoverpa zea*). Through choice assays and performance experiments, we demonstrate that salt‐stressed tomato plants are poor hosts for *H. zea*, negatively affecting caterpillar feeding preferences and growth rates. While changes in plant nutritional quality were observed, the primary factor influencing insect performance appears to be direct ionic toxicity, which significantly impairs multiple life history parameters of *H. zea* including survival, pupation, adult emergence, and fecundity. Plant defense responses show complex interactions between salt stress and herbivory, with two proteinase inhibitor genes ‐ *PIN2* and *AspPI*, showing a higher induced response to insect herbivory under salt conditions. However, plant defenses do not seem to be the main driver of reduced caterpillar performance on salt‐treated plants. Furthermore, we report reduced oviposition by *H. zea* moths on salt‐treated plants, which was correlated with altered volatile emissions. Our findings reveal that *H. zea* exhibits optimal host selection behaviours for both larval feeding and adult oviposition decisions, which likely contribute to its success as an agricultural pest. This research provides insights into the complex interactions between abiotic stress, plant physiology, and insect behaviour, with potential implications for pest management strategies in saline agricultural environments.

## Summary statement

We show that salt‐stressed tomato (*Solanum lycopersicum* cv. Better Boy) plants are poor hosts for the corn earworm (*Helicoverpa zea*) impacting caterpillar preference and performance, and moth oviposition.

## 1 Introduction

In their natural environment, plants frequently encounter two or more stresses that can limit their productivity and fitness (Holopainen and Gershenzon 2010; Spooner, Peralta, and Knapp 2005). These include both abiotic stresses (e.g., extreme temperatures, drought, water logging, and soil salinization) and biotic stresses (e.g., pathogen‐borne diseases and herbivory (insect or mammalian)) (Gull, Lone, and Wani 2019). However, the presence of an abiotic stressor can influence the impact of a biotic stressor. For example, elevated temperatures can predispose plants to pathogen infections (Scherm 1994), drought‐stressed plants can become susceptible to insect outbreaks (Lin et al. 2022), and soil salinization could drive variations in insect herbivory and herbivore demography (Marsack and Connolly 2022).

Soil salinization poses a major threat to global agriculture, with estimates suggesting that over 50% of cultivable lands will be salinized by 2050 (Ashraf 2009, 2010; Munns and Gilliham 2015; Zhao et al. 2020). Various natural and anthropogenic factors contribute to soil salinization, including low precipitation, saline groundwater contamination, poor irrigation, and seawater intrusion in coastal areas (Kaya et al. 2022; Sahbeni et al. 2023; Shrivastava and Kumar 2015; Y. Zhang et al. 2022). Salt stress impacts plants through osmotic stress, ionic toxicity by Na^+^ and Cl^‐^ ions that further disrupt osmotic balance, and oxidative damage (Munns and Tester 2008), collectively impairing photosynthesis and reducing plant growth and development. Consequently, this has been shown to significantly compromise plant yields in several crops such as rice, wheat, barley, and tomato (Parida and Das 2005; Shabala, Wu, and Bose 2015). A significant amount of the world's tomato produce is sourced from regions near the Mediterranean Sea, India, and California, areas characterized by their warm and arid climate, making them particularly prone to salinity (Cuartero and Fernández‐Muñoz 1998). Tomato plants, while moderately salt‐tolerant, experience reduced seed germination and fruit yields under high salinity conditions (Cuartero et al. 2006; Guo et al. 2022).

Biotic stresses such as insect herbivory can further reduce crop yields by influencing a plant's photosynthetic capacity, altering plant metabolism, nutrient acquisition, and allocation (Babst et al. 2008; Qu 2019). One such destructive agricultural pest, the tomato fruitworm caterpillar (*Helicoverpa zea*) causes annual losses exceeding $1 billion globally across various crops (Capinera 2000; da Silva et al. 2020).

Plants use distinct response pathways for individual stressors, but pathways can overlap and converge via shared signaling molecules or proteins (Sewelam, Kazan, and Schenk 2016). Upon herbivory, *H. zea* is known to trigger the jasmonate defense response pathway in plants, which induces the production of plant defense metabolites such as proteinase inhibitors (Bi, Murphy, and Felton 1997; Jiao et al. 2022; J. Wang et al. 2019). A study on salt stress in tomatoes reported that salt stress primed a jasmonate‐mediated anti‐insect herbivory defense response via ROS (reactive oxygen species) accumulation (Sabina and Jithesh 2021). Another study on tomatoes showed the accumulation of anti‐insect herbivory proteinase inhibitors under salt stress (Dombrowski 2003). A study on salt‐stressed maize showed decreased levels of anti‐insect herbivory metabolites (1,4‐benzoxazin‐3‐one‐glycones or aBX) (Forieri, Hildebrandt, and Rostás 2016). Elevated levels of plant defense metabolites can deter insect feeding and cause lower caterpillar growth rates (Divekar et al. 2022). As salt stress and herbivory co‐occur, plant responses to one stress can have implications for the other (Capiati 2006; Fujita et al. 2006). The specificity of plant responses to unique stress combinations hinders our comprehensive understanding of their complex interplay and emergent outcomes. It thus becomes difficult to predict how plants will respond to multiple stresses. In our study, by investigating insect performances and plant defenses under salinity and insect herbivory, we aim to provide a deeper understanding of how salinity and herbivory combinations affect tomato plants and herbivores feeding on them.

Furthermore, the performance of herbivorous pests feeding on stressed plants is influenced by changes to plant nutritional quality and defense chemical levels (Ali et al. 2021). While some studies demonstrate enhanced insect performances on salt‐stressed plants (Eichele‐Nelson et al. 2017; Han et al. 2016; Polack, Pereyra, and Sarandón 2011), others show decreased herbivore performances (Ali et al. 2021; Ghodoum Parizipour et al. 2021; Quais et al. 2019), with most studies on leaf‐chewing insects suggesting insects are deterred by salt‐stressed plant tissues (Martel 2013; Xiao et al. 2019; Dong et al. 2020). Insect herbivore performance on salt‐stressed plants depends upon the specific plant variety and insect species under consideration (Awmack and Leather 2002; Huberty and Denno 2004). For example, soybean looper (*Pseudoplusia includens*) growth rate was reduced on a salt‐sensitive soybean variety but remained unchanged on a salt‐tolerant variety (Najjar et al. 2018). Very few studies attribute lower insect performances on salt‐stressed plants to ionic toxicity in insects (Emery et al. 1998; Prather et al. 2018). The accumulation of NaCl in plant tissues, followed by its ingestion, could be toxic to insects, and has been shown to interfere with neural and muscle development (Snell‐Rood et al. 2014; Xiao et al. 2010). Insects would also expend considerable amounts of energy to maintain cellular Na^+^ levels, leading to increased metabolic demands, resulting in lower insect growth (Hund et al. 2023).

The establishment of insect pests on a plant usually begins with an adult moth choosing appropriate hosts via volatile organic compound (VOCs) cues and laying its eggs on the host plant (Karban 2008; Ninkovic, Markovic, and Rensing 2021; Peñaflor et al. 2011). Ovipositional decisions by moths determine where their larvae will feed and thus dictate larval performance on host plants. Plants, in a bid to conserve water under salt‐stress, reduce transpiration by closing their stomata, which in turn impacts plant VOC emissions and profiles (Forieri, Hildebrandt, and Rostás 2016; Lin et al. 2022; Munns and Tester 2008). Therefore, along with assessing caterpillar performance on plants, it becomes necessary to investigate moth oviposition preferences and VOC profiles to get a comprehensive idea of the plant‐insect interaction at play.

In this study, we investigated the effects of salt stress (NaCl) on the nutritional quality of tomato plants (*Solanum lycopersicum*) and their resistance to herbivory by the generalist polyphagous tomato fruitworm caterpillar *Helicoverpa zea* Boddie (Lepidoptera: Noctuidae) (Fitt 1989; Quaintance and Brues 1905). We hypothesized that salt‐treated tomato plants would be poor hosts for *H. zea*, negatively affecting caterpillar feeding preferences, growth rates, and moth oviposition (Renault et al. 2016). Our aim was to disentangle various mechanisms by which *H. zea* caterpillars could be affected by salinity stress: (i) through direct ionic toxicity, (ii) through altered plant nutritional quality, and/or (iii) through increased plant defense chemicals. We examined the impact of ionic toxicity on various *H. zea* life history parameters alongside changes to plant water content and nutritional quality. We evaluated anti‐herbivore defenses by analyzing defense gene expression, defense protein levels, and volatile emissions. We quantified herbivore‐induced defense responses in control and salt‐stressed plants to determine how biotic and abiotic stressors interact to influence plant resistance. Lastly, we investigated *H. zea* moth oviposition preferences and collected volatile compounds from control and salt‐treated plants.

## 2 Materials and Methods

### 2.1 Plants

Tomato plants *Solanum lycopersicum* cv. Better Boy (BB) (Harris Seeds, Rochester, NY, USA) were transplanted post‐germination and grown in cubic plastic pots (3.5″ x 3.5″ x 3.5″) filled with Metromix 400 potting mix (Griffin Greenhouse & Nursery Supplies, Tewksbury, MA, USA) in a greenhouse at the Pennsylvania State University (25 ± 2°C, 70% ± 10% R.H., 16 L: 8D). Plants were fertilized at the one‐leaf stage with Osmocote Plus 15‐9‐12 Fertilizer (ICL Speciality Fertilizers, Summerville, SC, USA). Tomato plants at the four‐leaf stage (4 weeks old) were used in all experiments.

Our study focused primarily on the most prevalent and well‐studied salt, NaCl (sodium chloride) (Munns and Tester 2008; Rengasamy 2010). Plants were subjected to two salt treatments:

1. No salt/untreated control (0 mM): 200 mL of a 0 mM salt solution (distilled water) was added to the base of the plant.
2. Salt‐treated (200 mM): 200 mL of a 200 mM salt solution was added to the base of the plant.

200 mM of NaCl was the chosen concentration based on multiple studies (Dombrowski 2003; Foolad 1996; Forieri, Hildebrandt, and Rostás 2016; Munns and Gilliham 2015; Sun et al. 2010; H.‐X. Zhang and Blumwald 2001). Plants were watered with their designated salt solutions every day for 3 days unless specified otherwise, following which they were used for experiments on the third day. It is worth noting that at the end of the 3‐day period, the soil salt concentration might be higher than the supplied amount due to salt accumulation in the soil. Plant parameters such as plant fresh weight (g), stomatal conductance ((LI‐600 handheld fluorometer ‐ (LI‐COR Inc.), and chlorophyll content (Leaf analyzer Dualex ‐ (Dualex Scientific Chlorophyll Meter, FORCE‐A, Orsay, France)) were also measured at the end of 3 days (Figure S1).

### 2.2 Insects

Tomato fruitworm *(Helicoverpa zea)* eggs were purchased from Benzon Research (Carlisle, PA USA) and caterpillars were reared on a wheat‐germ‐based artificial diet (Peiffer and Felton 2005) until pupation. Adult moths were provided with a 10% sugar solution, and eggs were collected daily to be used in the experiments.

### 2.3 Caterpillar Preference Assay

To determine caterpillar preference for plant tissue with various levels of salt stress, we conducted a two‐choice assay between a no salt‐treated plant leaf disc (0 mM) vs a salt‐treated plant leaf disc (200 mM) (Pan et al. 2019).

1.5‐cm‐diameter leaf discs were excised from the fourth fully expanded leaf of each treatment plant. One leaf disc from each treatment was placed at opposite ends of a Petri dish (10 cm × 1.5 cm) lined with 3% agar. A single 2nd instar *H. zea* caterpillar was placed in the middle of the Petri dish, and the Petri dishes were sealed with parafilm to prevent the desiccation of herbivores and plant tissue (Marsack and Connolly 2022). Caterpillars were given 30 min to make a choice in the Petri dish, after which the leaf disc the caterpillar had settled on was recorded. This was referred to as the “first establish” parameter. Another parameter called the “first finish” was reported as the first leaf disc to be totally consumed by the caterpillar (Figure S2). First finishes were recorded for a maximum of 5 days, following which the experiment was concluded. After 2 days of feeding, leaf area consumed or caterpillar consumption rate was determined by photographing the remaining leaf disc area and analyzing images in ImageJ (Connolly, Guiden, and Orrock 2017; Glozer 2008; Marsack and Connolly 2022; Orrock et al. 2018). Thirty‐six Petri dishes (feeding arenas) were set up in this experiment (*n* = 36).

### 2.4 Caterpillar Performance Assay

To determine *H. zea* caterpillar relative growth rate (RGR) under different salt regimes, three no‐choice feeding assays were conducted:

1. Detached leaf assay: The fourth fully expanded leaf from each no salt‐treated and salt‐treated plants was excised and placed in individual plastic cups lined with 3% agar (to ensure the leaves retained moisture). A single pre‐weighed 2nd instar *H. zea* caterpillar was added to each cup. Caterpillars were weighed again after 3 days of feeding (*n* = 80 per treatment).
2. Whole plant assay: No salt‐treated and salt‐treated plants were placed in individual cages, and a single pre‐weighed 2nd instar *H. zea* caterpillar was placed on the plant. The insect was allowed to feed freely on the plant, following which it was weighed again after 3 days of feeding (*n* = 42 per treatment).
  Caterpillar relative growth rate (RGR) was calculated as:
  RGR = (W2−W1)/(W1 × (T2−T1)), where W1 and W2 are caterpillar weights at time T1 and T2, respectively.
3. Artificial insect diet‐based dose‐response assay: To evaluate fitness costs of salt concentration on insects, artificial diet was spiked with multiple salt concentrations (0, 50, 100, 200, 400, 800, and 1600 mM). The final concentration of Na^+^ in 0 mM artificial diet was 875 mg/kg and in 200 mM artificial diet was 4560 mg/kg. All diets were made using serial dilutions and the concentrations of Na^+^ in other diets can be extrapolated from the values for 0 mM and 200 mM diets. A piece of artificial diet (1 × 1 × 1 cm) was placed in a caterpillar‐rearing cup, following which one 2nd instar *H. zea* caterpillar was added to each cup (*n* = 60 per treatment). Over the next few weeks, the following insect growth parameters were recorded: caterpillar mortality, average number of days required to pupate, pupal weight (mg), average number of days spent as a pupa, adult emergence %, and adult wing deformation % (Figure S3). Caterpillar photographs were taken after 7 days of feeding. Female pupal weight was used as a proxy for female fecundity (Tisdale and Sappington 2001).

### 2.5 Quantifying Plant Nutritional Quality

Plants treated with 0 mM and 200 mM for 3 days were dried and ground, following which they were sent to the Penn State Analytical Laboratory for ion content/nutrient analysis (P, K, Ca, Mg, S, Mn, Zn, Cu, B, Al, Fe, and Na contents – Table S1) (C. L. Huang and Schulte 1985) (*n* = 6 per treatment). Plant relative water content and total protein content were also measured (Notes S1, Figure S4) as a measure of plant quality.

Average Na^+^ levels were also measured in caterpillar frass to investigate insect regulation of Na^+^ (Notes S3).

### 2.6 Caterpillar Performance Assay on Salt‐Stress and Herbivore‐Induced Plant Leaves

A two‐factorial assay with Salt (No salt and Salt) and Herbivory (No herbivory and Herbivory) as factors was conducted. For the Herbivory treatment, a single, 1‐day starved 5th instar *H. zea* caterpillar was allowed to feed inside a clip cage (3.15 cm^2^ leaf area) on the fourth fully expanded leaf of a tomato plant. The clip cage and larva were removed once the leaf inside the clip cage was completely consumed. Forty‐eight hours later, the remaining part of the tomato leaf was fed to insect herbivores in a cup‐based assay referred to as the “Herbivory” treatment (these leaf tissues have induced their chemical defenses due to insect herbivory) (Paudel et al. 2019). Empty clip cages were placed on plants in the “No herbivory” treatment. No salt and Salt treatment leaves were excised from no salt‐treated and salt‐treated plants. A single pre‐weighed 2nd instar *H. zea* caterpillar was added to each cup. Caterpillars were weighed again after 3 days of feeding (*n* = 30 per treatment).

### 2.7 Plant Defense Responses

#### 2.7.1 Gene Expression

To determine the induction of tomato defense responses under joint salt stress and insect herbivory, the expression of defense‐related genes such as *PIN2* (proteinase inhibitor II), *ASPPI* (aspartic proteinase inhibitor), *PPOB* (polyphenol oxidase), *TD2* (threonine deaminase), and *CYSPI* (cysteine proteinase inhibitor) was measured (Dombrowski 2003; Tan et al. 2018).

A two‐factorial assay with Salt (No salt and Salt) and Herbivory (No herbivory and Herbivory) was conducted. Following salt treatment for 3 days, a single, 1‐day starved 5th instar *H. zea* caterpillar was allowed to feed inside a clip cage (3.15 cm^2^ leaf area) on the fourth fully expanded leaf of the plant. The clip cage and larva were removed once the leaf inside the clip cage was completely consumed. Twenty‐four h later, treated leaves were flash frozen in liquid nitrogen and stored at −80°C until further analysis. Empty clip cages were placed on plants in the “No herbivory” treatment.

For quantitative real‐time polymerase chain reaction (qRT‐PCR), leaves were homogenized in a Geno Grinder 2000 (OPS Diagnostics, USA), and total RNA was purified using TRIZOl. 1 μg of purified RNA was transcribed to cDNA using a High‐Capacity cDNA Reverse Transcription Kit (Applied Biosystems, USA). For qRT‐PCR primers refer to Table S3. qRT‐PCR reactions using Power‐Track SYBR Green PCR Master Mix (Applied Biosystems, USA) were run on a 7500 Fast Real‐Time PCR System (Applied Biosystems, USA) using a previous protocol (Acevedo et al. 2017).

Relative quantification of genes was calculated using the 2^−ΔΔ*C*^_T_ method (Livak and Schmittgen 2001), with No salt x No herbivory‐treated plants as the reference group and ubiquitin as a housekeeping gene to normalize C_T_ values (Rotenberg et al. 2006) (*n* = 5 – 7 per treatment).

#### 2.7.2 Defense Proteins

To investigate the interactive effect of salt stress and insect herbivory on tomato defense responses, the activity of two jasmonic acid (JA)‐inducible plant defense proteins: polyphenol oxidase (PPO: mOD/min/mg tissue) and trypsin proteinase inhibitor (TPI: % inhibition/mg protein) were measured (Tan et al. 2018) (Notes S2, Figures S5, and S6).

### 2.8 Moth Oviposition Assay

Gravid female *H. zea* moth oviposition preferences were tested for tomato plants with differing salt exposures (no salt and salt). In a butterfly‐rearing cage (24″ × 24″ × 36″), no salt‐treated and salt‐treated plants of similar heights were placed 30 cm apart at opposite ends of the cage. Three pairs of *H. zea* moths, mated for 24 h prior to the assay were released in the cage. The total number of eggs on each plant was recorded after 1 day (Paudel et al. 2019). A 10% sucrose solution was provided to the moths for feeding during the assay. Dead moths were replaced as needed throughout the experiment, although this was only for 7 out of the total 41 replicates (*n* = 41). The plant arrangement inside the cage was randomized to counter location biases in the setup.

### 2.9 Volatile Organic Compound (VOC) Collection and Analysis

Individual tomato plants from each salt treatment (*n* = 10 plants per treatment) were placed in a 9‐liter glass chamber, and VOCs were collected for 12 h using a push‐pull system at a push flow rate of 1 L/min and a pull flow rate of 0.8 L/min, following which plant fresh shoot weights (FW) were recorded. VOCs were collected using adsorbent filters containing 45 mg HayeSep Q (Hutchison Hayes Separation Inc. USA) and were eluted by passing 150 μL dichloromethane through the traps into 2.0 mL glass vials equipped with a 250 μl glass insert. 5 µL of internal standards octane (40 ng µl^‐1^) and nonyl acetate (40 ng µl^‐1^) were added to the samples (Helms et al. 2019).

VOCs were analyzed by GC‐MS using an Agilent ‐ 7890 A gas chromatograph equipped with an Agilent HP‐5MS UI (30 m × 0.25 mm × 0.25 µm) column coupled to an Agilent ‐ 5975 C mass spectrometer configured with standard EI tune settings. Splitless injections were performed at an inlet temperature of 250°C using helium carrier gas with a constant flow rate of 0.7 mL/min. After 1 µL sample injection, the column was maintained at 40°C for 2 min. The oven temperature was increased by 10°C per minute and held at 300°C for 4 min. Target compounds were identified by comparing mass spectra and retention indices published in NIST17, Adams, and the University of Göteborg libraries (Helms et al. 2017; Lin et al. 2021). Compounds with quality scores > 85 were selected for further analysis. The abundance of each compound per gram of fresh plant tissue was calculated using the internal standard of nonyl‐acetate (Table S2).

### 2.10 Statistical Analysis

Data were assessed for normality, and non‐normal data was transformed using natural logarithms or square root transformations when necessary. If data transformations were unsuccessful, non‐parametric tests were used to determine significance. Caterpillar percentage leaf area consumption was compared using paired T‐tests between the treatments for the two‐choice assays. Caterpillar first finishes and establishes were compared using Chi‐square tests with a 1:1 choice assumption. Unpaired Student's T‐test was used for caterpillar RGR, water content, protein content, ionic content, plant nutrient content analysis, and for individual volatile compound emissions. A non‐parametric Kruskal‐Wallis test followed by Dunn's multiple comparisons was used for all salt‐spiked artificial diet results. A mixed‐effects model was used to analyze gene expressions. Differences in the average eggs laid on no salt‐treated and salt‐treated plants were compared using a two‐way ANOVA (repeated measures model). Volatile emission differences under salt treatment were analysed using a permutational multivariate analysis of variance (PERMANOVA, with Salt x Herbivory as factors). A principal component analysis (PCA) was performed on the analyzed compounds for visual representation.

## 3 Results

### 3.1 Plant Parameters

By the end of the 3‐day treatment, salt‐treated plants appeared visibly stressed, showed reduced growth, lower stomatal conductance, and were darker in colour with a higher chlorophyll content (Figure S1).

### 3.2 Caterpillar Preference Assay

**Figure 1:**
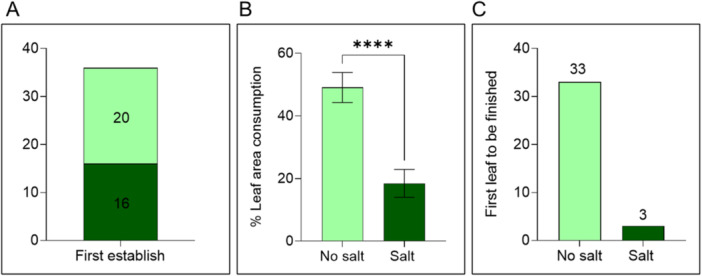
Caterpillar preference assay. Petri dish‐based two‐choice *H. zea* feeding preference assay on tomato plant leaf discs treated with varying levels of salinity. (A) First leaf that a caterpillar is recorded on after 30 min (first establish). (B) Percentage leaf area consumed after 48 h of caterpillar feeding. Bars represent mean (± SEM) (*n* = 36, Student's *T*‐test, **** *p* < 0.0001). (C) First leaf to be finished in a dual choice given to insect caterpillars (first finish).

In Petri dish‐based two‐choice leaf disc assays, *H. zea* caterpillars did not preferentially feed or establish on any leaf tissue at first with 20 caterpillars choosing no salt‐treated leaf discs and 16 caterpillars choosing salt‐treated leaf discs (Figure 1A; First establish (FE): χ² = 0.444, df = 1, *p* = 0.5050), however, they showed differences in feeding over time. Percentage leaf area consumed by *H. zea* caterpillars was significantly higher in no salt‐treated (0 mM) leaf discs compared to salt‐treated leaf discs (200 mM) (Figure 1B; *t* = 4.861, df = 34, *p* < 0.0001). Caterpillars completely consumed leaf discs treated with 0 mM salt first (first finish) (Figure 1C; χ² = 25, *p* < 0.0001; Figure S2).

### 3.3 Caterpillar Performance Assay

**Figure 2:**
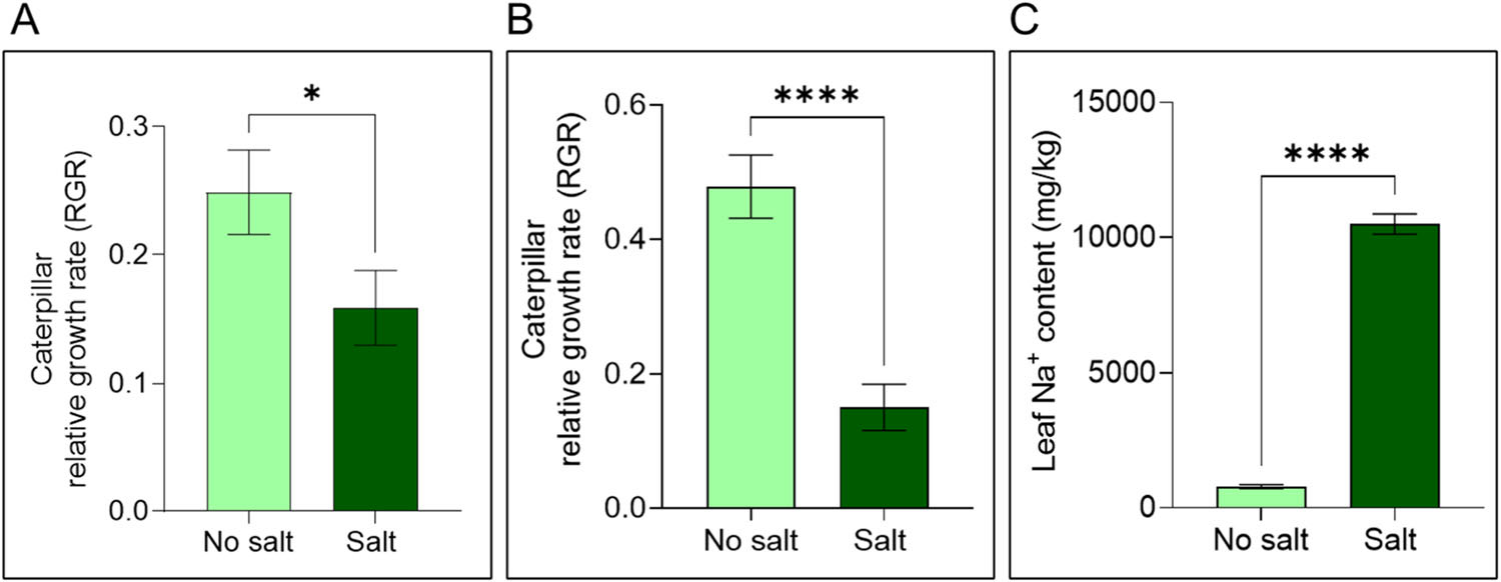
Caterpillar performance assay. (A) Detached‐leaf assay caterpillar RGR estimation. (B) Whole plant‐based caterpillar RGR estimation. (C) Leaf Na^+^ content (mg/kg). Bars represent mean (± SEM) (A: *n* = 80; B: *n* = 42; C: *n* = 6; Student's *T*‐test, * *p* < 0.05, **** *p* < 0.0001). [Color figure can be viewed at wileyonlinelibrary.com]

*H. zea* caterpillar relative growth rate (RGR) was higher on no salt‐treated plants compared to salt‐treated plants in a detached leaf assay (Figure 2A; *t* = 1.983, df = 158, *p* < 0.05) and in a whole‐plant assay (Figure 2B; *t* = 5.588, df = 81, *p* < 0.0001). Salt addition significantly increased leaf Na^+^ content from 786.8 ± 63.83 mg/kg in 0 mM leaves to 10497 ± 374.8 mg/kg in 200 mM leaves (Figure 2C; *t* = 25.54, df = 10, *p* < 0.0001).

### 3.4 Artificial Insect Diet Dose Response With Salt Concentration Assay

**Figure 3:**
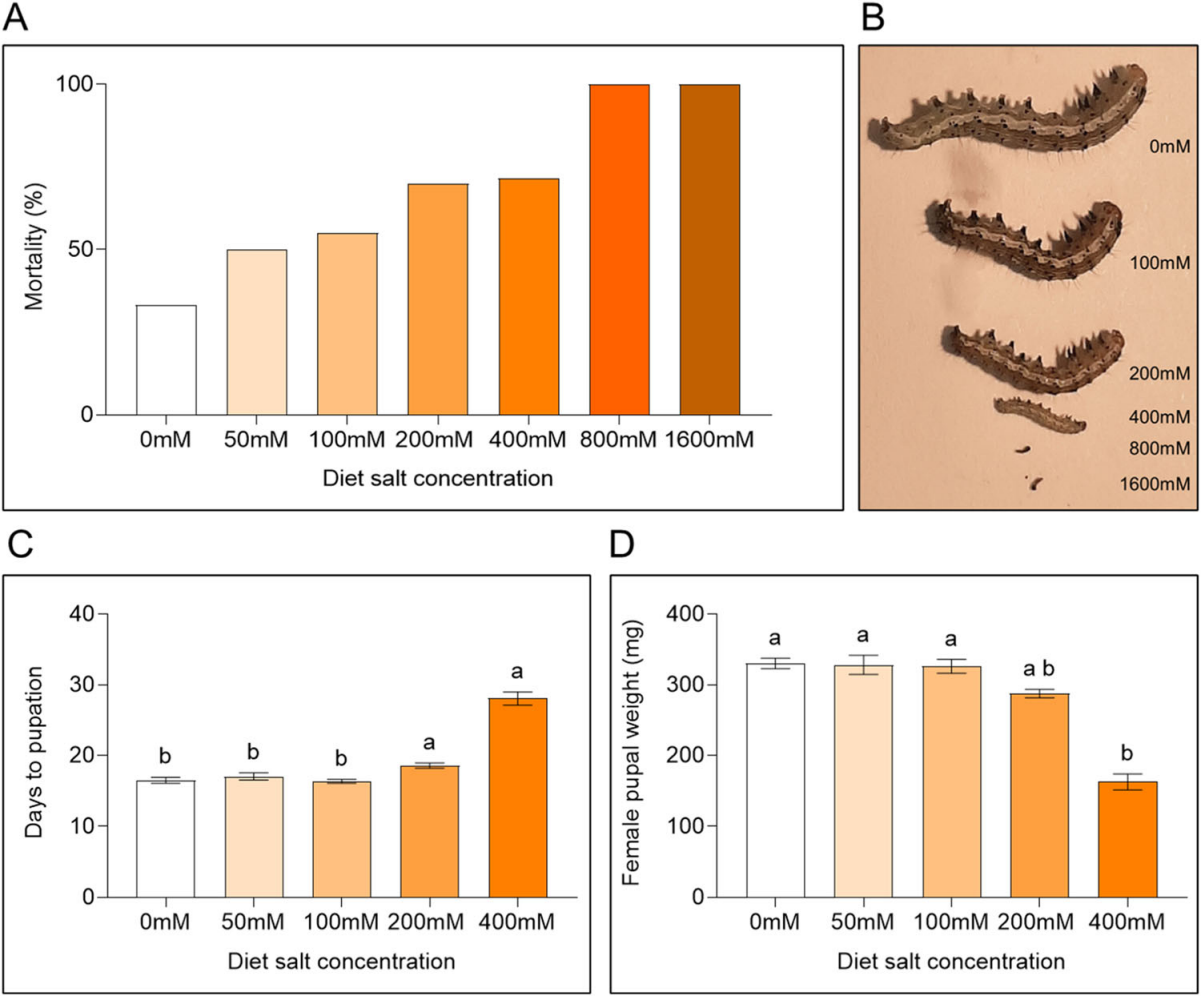
Artificial insect diet dose response with salt concentration assay. (A) Insect mortality at various salt concentrations. (B) Caterpillar sizes after 7 days of feeding on artificial diet. (C) Average number of days required to reach pupation by caterpillars. (D) Average female pupal weight (mg). Bars represent mean (± SEM) (*n* = 60, different letters indicate significant differences *p* < 0.05). [Color figure can be viewed at wileyonlinelibrary.com]

To investigate the effect of ionic toxicity on caterpillar growth parameters, we conducted a salt‐spiked artificial insect diet‐based assay. *H. zea* mortality increased with salt treatment (33.33% in 0 mM, 50% in 50 mM, 55% in 100 mM, 70% in 200 mM, and 71.66% in 400 mM) (Figure 3A). Insects fed on 800 mM and 1600 mM salt‐spiked diet showed 100% mortality, and hence adult parameters are not shown. Caterpillar growth was affected by salt treatment (Figure 3B). The average number of days required by caterpillars to reach pupation remained unchanged within 0 mM (16.53 ± 0.4145 days), 50 mM (17.03 ± 0.4948 days), and 100 mM (16.37 ± 0.2623 days) diets, but were significantly more on 200 mM (18.56 ± 0.3811 days) and 400 mM (28.06 ± 0.9412 days) diets (Figure 3C; Kruskal–Wallis statistic = 61.01, df = 5, *p* < 0.0001). Average female pupal weight remained unchanged between 0 mM (330.3 ± 7.295 mg), 50 mM (328.3 ± 13.51 mg), 100 mM (326.3 ± 9.843 mg), and 200 mM (287.7 ± 6.011 mg) diets, but significantly decreased on 400 mM (162.9 ± 11.12 mg) diet (Figure 3D; Kruskal–Wallis statistic = 59.53, df = 5, *p* < 0.0001). Average days to adult emergence from pupae remained unchanged between treatments, % moth emergence reduced, and % wing deformation increased as the salt concentration increased (Figure S3).

### 3.5 Quantifying Plant Nutritional Quality

Salt‐treated plants showed significantly lower relative water contents and lower leaf total protein contents as compared to no salt‐treated plants (Figure S4). Salt‐treated plants showed altered ion contents/nutrient levels as compared to no salt‐treated plants, with significant differences observed in Ca, Mg, S, Mn, Zn, Cu, and B levels (Table S1).

### 3.6 Caterpillar Performance Assay on Salt‐Stress and Herbivore‐Induced Plant Leaves

**Figure 4:**
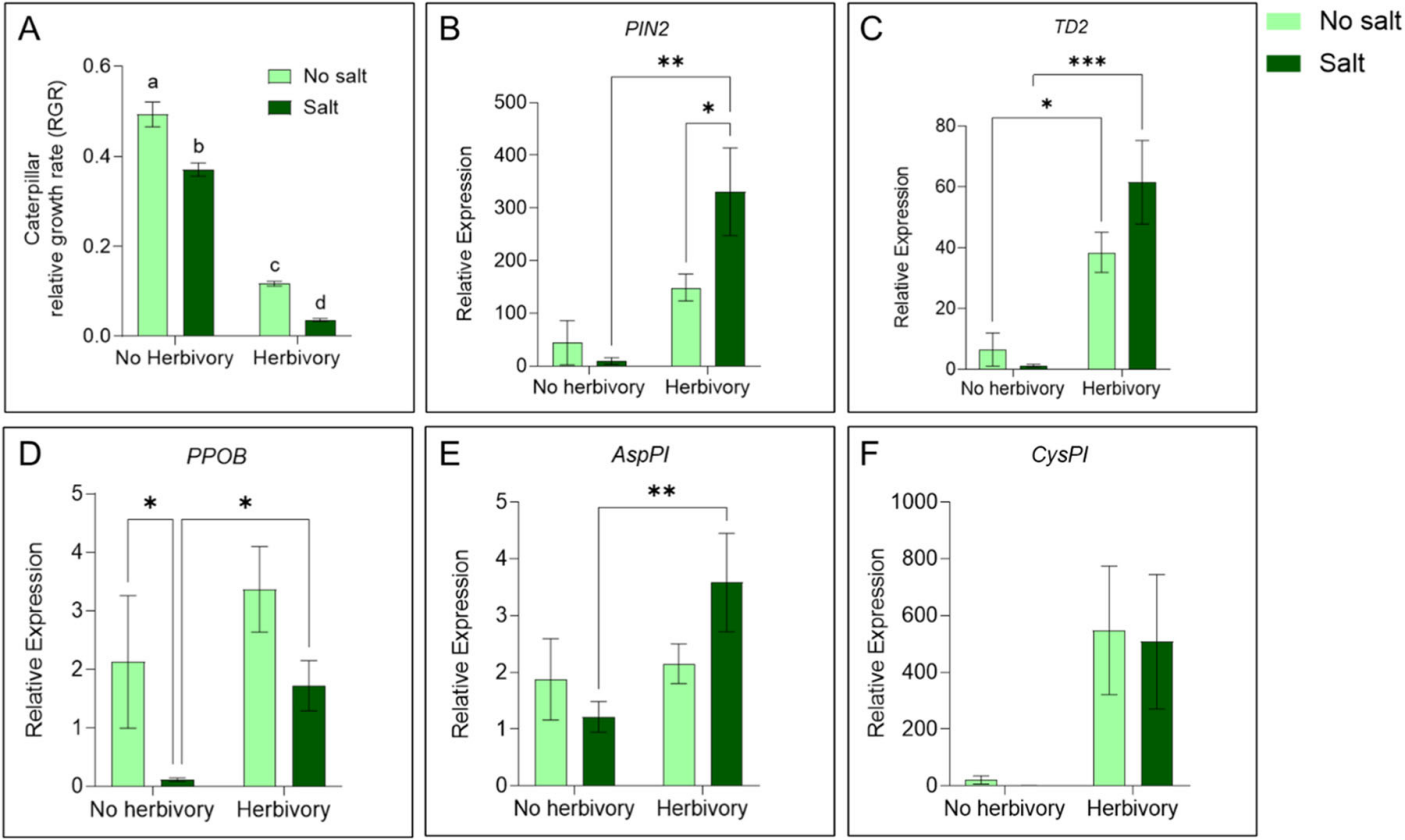
Caterpillar performance assay on salt‐stress and herbivore‐induced plant leaves and plant defense gene expression responses. (A) *H. zea* caterpillar RGR feeding on (No salt, Salt) x (No herbivory, Herbivory) plant tissue in a cut‐leaf agar cup‐based assay. Bars represent mean (± SEM) (*n* = 30, Two‐way ANOVA, different letters indicate significant differences: *p* < 0.05). Plant defense and secondary metabolite synthesis gene levels (B) *PIN2*: proteinase inhibitor II; (C) *TD2*: threonine deaminase; (D) *PPOB*: polyphenol oxidase B; (E) *AspPI*: aspartic proteinase inhibitor; (F) *CysPI*: cysteine proteinase inhibitor in plants treated with Salt and Herbivory (No herbivory and Herbivory) treatments. Bars represent mean ± SEM expression relative to no herbivory, no salt control (*n* = 5–7, mixed‐effects analysis, * *p* < 0.05, ** *p* < 0.01, *** *p* < 0.001, **** *p* < 0.0001). [Color figure can be viewed at wileyonlinelibrary.com]

There was a significant main effect of Salt (F_(1, 58)_ = 35.93, *p* < 0.0001), and Herbivory (F_(1, 56)_ = 557.9, *p* < 0.0001) on caterpillar RGR, but no significant interaction between Salt and Herbivory (F_(1, 56)_ = 1.957, *p* = 0.1673). Insect growth rate reduced by feeding on defense‐induced plant parts. Salt and Herbivory have additive negative effects on caterpillar growth (Figure 4A).

### 3.7 Plant Defense Responses

#### 3.7.1 Gene Expression

There was a significant main effect of Herbivory for all genes, while there was a significant main effect of Salt only for *PPOB*, with a significantly lower *PPOB* gene expression in the salt treatment (Figure 4D, Table S4). In undamaged plants (no herbivory), all genes except *PPOB* showed equal gene expression between the no salt and salt treatment. In herbivore‐damaged plants, *PIN2* and *AspPI* show a higher induced gene expression in the salt treatment compared to the no salt treatment (Figure 4B,E). Refer to Table S4 for mixed‐effects model results.

#### 3.7.2 Defense Proteins

Salt addition alone did not lead to an increase of PPO and TPI levels in tomato plants (Figure S5). Salt addition over short (6 h, 24 h) and long (3 days, 7 days) durations does not prime plants for higher induced PPO and TPI response to insect herbivory (Figure S6).

#### 3.7.3 Moth Oviposition Assay

**Figure 5:**
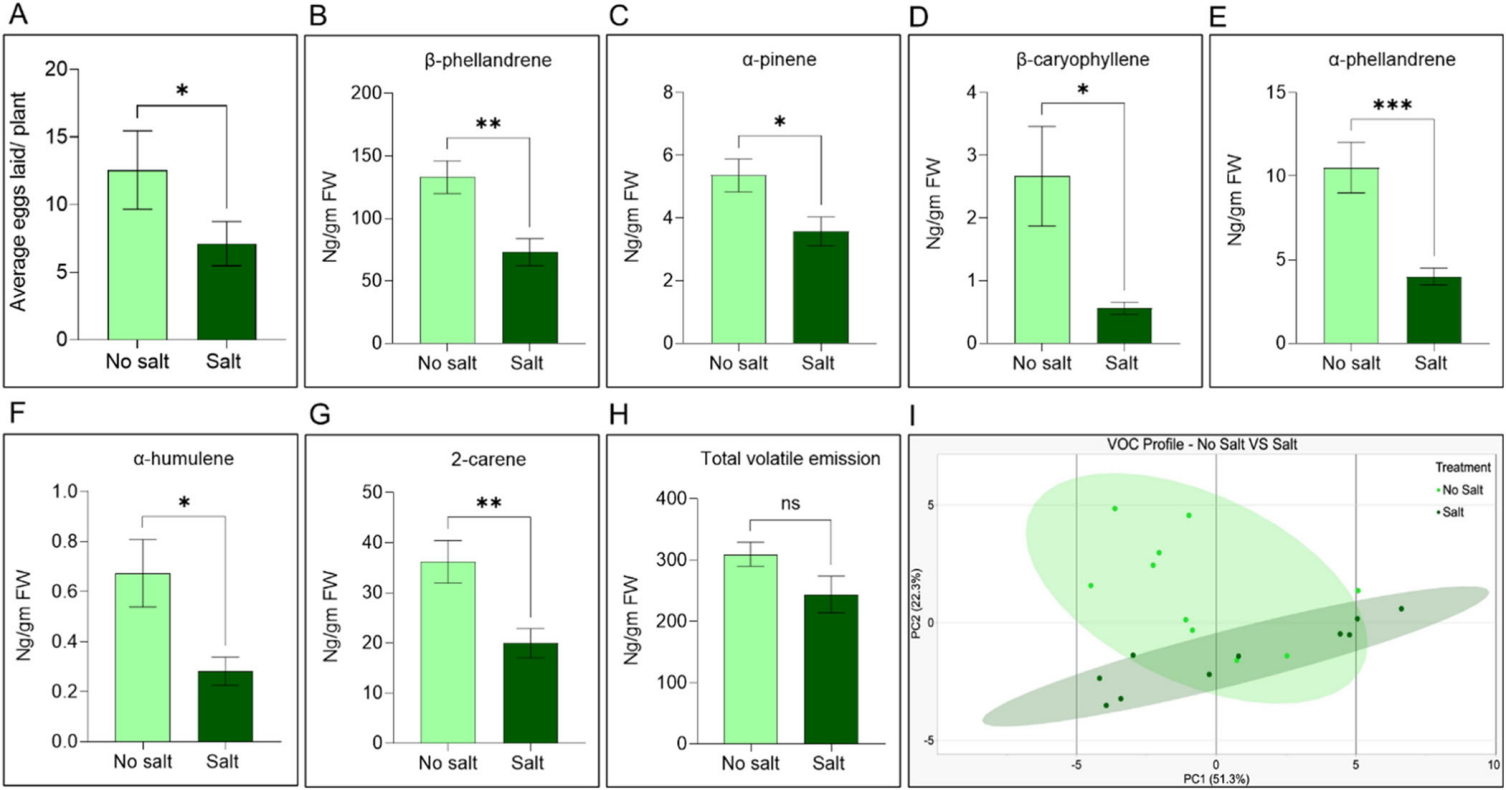
Moth oviposition assay and VOC analysis. (A) Female *H. zea* moth oviposition preference on no salt and salt‐treated plants. Bars represent mean (± SEM) number of *H. zea* eggs laid/plant (*n* = 41, Students *T*‐test, * *p* < 0.05). (B–G) Differences in specific VOC emission (ng/g fresh weight) by tomato plants under no salt and salt treatments. Bars represent mean VOC emission (± SEM) (*n* = 10, Students T‐test, * *p* < 0.05, ** *p* < 0.01). (H) Total volatile emission (ng/g fresh weight) of no salt and salt‐treated plants. (I) PCA plot of the volatile blend from no salt and salt‐treated plants. [Color figure can be viewed at wileyonlinelibrary.com]

Gravid female *H. zea* moths showed a significant oviposition preference for no‐salt‐treated plants compared to salt‐treated plants. After 1 day, the average number of eggs laid on no salt‐treated plants (12.56 ± 2.894) was significantly higher than the average number of eggs laid on salt‐treated plants (7.122 ± 1.641) (Figure 5A; *t* = 2.045, df = 40, *p* = 0.0475).

### 3.8 Volatile Organic Compound (VOC) Analysis

Even though the emission of specific VOCs (Table S2) like β‐phellandrene (Figure 5B; *t* = 2.571, df = 18, *p* = 0.0192), α‐pinene (Figure 5C; *t* = 2.559, df = 18, *p* = 0.0197), β‐caryophyllene (Figure 5D; *t* = 2.637, df = 18, *p* = 0.0167), α‐phellandrene (Figure 5E; *t* = 3.514, df = 18, *p* = 0.0025), α‐humulene (Figure 5F; *t* = 2.504, df = 18, *p* = 0.0221), and 2‐carene (Figure 5G; *t* = 2.43, df = 18, *p* = 0.0258) decreased post salt treatment, total volatile emission per biomass (ng/g FW) remained unchanged (Figure 5H; *p* > 0.05). The overall blend of the plant volatiles was significantly different between no salt and salt treatments (Figure 5I; PERMANOVA (F_(1, 9)_ = 3.546, *p* = 0.0069).

## 4 Discussion

In this study, we showed that tomato fruitworm (*Helicoverpa zea*) insects showed reduced establishment and performance on salt‐treated tomato plants. The negative *H. zea* response to salt‐treated tomato plants seems to be primarily caused by direct ionic toxicity challenging traditional focus on changes in plant nutritional quality and plant defensive compounds as the main mediators of plant‐insect interactions under stress conditions. The observed plant defense responses further suggest a complex interplay between abiotic stress and plant defenses. Pest performances seem to reduce under conditions of high salinity and can help dictate pest management strategies in saline agricultural environments.

### 4.1 Plants Grown Under Salt Stress Impact Caterpillar Leaf Disc Preference and Growth Adversely

In Petri dish‐based two‐choice assays, caterpillars initially showed no preference (first establish—Figure 1A) but gradually favored no salt‐treated leaf discs over salt‐treated leaf discs. This preference was evident both in percentage leaf area consumption (Figure 1B) and the first finish parameter (Figure 1C, Figure S2). Although caterpillars can be attracted to plants by volatile or visual cues (Perkins et al. 2008; von Mérey et al. 2013), in our study, caterpillar behavior suggests that they assess leaf quality through feeding before establishing a preference.

Some previous studies have also reported insect preference for plants under lower salinity conditions (Ali et al. 2021; Marsack and Connolly 2022; Quais et al. 2020). Herbivores are known to preferentially feed on plants or plant parts that are more nutritious and ensure their survival (Price 1991). We observed that caterpillar feeding preference was complemented by caterpillar growth rates. In no‐choice assays using both detached leaves and whole plants, *H. zea* caterpillars had reduced growth rates on salt‐treated plant tissue (Figure 2A,B). This finding aligns with a study on fall armyworm that showed reduced performance on salt‐stressed maize (Z.‐L. Wang, Haseeb, and Zhang 2021). Several factors, such as plant water content, nutritional quality (as measured via total protein content and plant nutrients), ionic toxicity, and levels of anti‐insect herbivore defense proteins may collectively contribute to reduced *H. zea* caterpillar performance on salt‐stressed plants (Felton 1996).

Salt stress led to a reduction in leaf water content and decreased total protein contents (Figure S4) (D. Coley, L. Bateman, and A. Kursar 2006). While it is well known that insects grow better on plants with a higher water and protein content (Cuartero and Fernández‐Muñoz 1998; Najjar et al. 2018; Z.‐L. Wang, Haseeb, and Zhang 2021), a study on alfalfa reported that salt‐stress‐based protein content reduction was not linked to altered insect performance on plants (Lei et al. 2017).

To assess plant nutritional quality, we measured plant nutrients such as P, K, Ca, Mg, S, Mn, Zn, Cu, B, Al, and Fe (Table S1). We observed significantly higher levels of Ca, Mg, Mn, and Zn, and significantly lower levels of S, Cu, and B in salt‐treated plants compared to no salt‐treated plants (Table S1). Although significant, these changes are relatively small in magnitude and may not directly influence caterpillar growth and development. However, these alterations indicate plant responses to salt stress. For example, Mn, Zn, and Cu are key components of superoxide dismutases; reactive oxygen species (ROS) scavenging enzymes that convert superoxide radicals into H_2_O_2_ (Hernández‐Nistal, Dopico, and Labrador 2002; Wu et al. 1999). The observed accumulation of Mn and Zn might suggest the presence of ROS molecules and oxidative stress in plant tissues (as expected with salt‐treatment) (Miller et al. 2010). Ingestion of ROS from plants could induce oxidative stress in caterpillars delaying their development (Apirajkamol et al. 2020). We did not directly measure ROS accumulation or damage in plant or insect tissue in our study.

In a no‐choice setup, we observed that caterpillars consumed a lower amount of salt‐treated leaf tissue compared to untreated tissue (unpublished results). Caterpillars can compensate for lower water content and nutrition by consuming a higher amount of tissue (Lee 2017). However, we did not observe any compensatory feeding, indicating the presence of feeding deterrents present in plant tissue. Given that changes in plant nutritional quality were insufficient to fully explain the observed effects on caterpillars, we focused on investigating ionic toxicity and plant defenses as feeding deterrents.

### 4.2 Ionic Toxicity Due to Increased Salt Concentration Directly Impacts Caterpillar Growth

We detected an accumulation of Na^+^ ions in salt‐stressed tomato plant leaves (Figure 2C) (H.‐X. Zhang and Blumwald 2001). To isolate the effect of ionic toxicity, we conducted an artificial diet dose–response experiment with varying salt concentration. As the concentration of salt in the artificial diet increased, *H. zea* growth and survival parameters were negatively affected. Salt negatively impacted caterpillar survival, growth, pupation, moth emergence, moth wing formation, and moth fecundity (Figure 3; Figure S3). Similar results have been reported in *Helicoverpa armigera* and *Danaus plexippus* (monarch butterflies) (Hund et al. 2023; Xiao et al. 2010). Although many insects seek Na^+^ actively, they usually require trace amounts of Na^+^ and high concentrations are detrimental to insect growth, disrupting the insect's osmotic balance, inducing oxidative stress, and interfering with the excretory, digestive, and respiratory systems (Martel 2013; Silver and Donini 2021). The neural and muscle development of monarch butterflies is negatively affected by high levels of Na^+^ (Snell‐Rood et al. 2014). We saw elevated Na^+^ levels in insect frass under salt treatment (Notes S3). Regulating Na^+^ levels and their excretion is metabolically expensive, which may explain why caterpillars grew slowly on salt‐treated diets (Hund et al. 2023). These results strongly suggest that ionic toxicity due to increased salt concentration directly impacts caterpillar growth and development, although the specific mechanisms are yet to be investigated.

### 4.3 Plant Defense Responses

Our investigation into plant defense responses revealed complex interactions between salt stress and herbivory. To tease apart the influence of ionic toxicity and plant defense chemicals, we conducted a detached‐leaf assay combining salt treatment with prior insect herbivory. We saw that salt and prior insect herbivory have additive negative effects on caterpillar growth (Figure 4A).

Salt treatment alone did not induce higher levels of plant defense genes (except *PPOB*) or proteins or increase plant inducibility to insect herbivory (Figure 4B–F, Figures S5 and S6). However, under combined salt and insect herbivory, *PIN2* (Proteinase Inhibitor II) and *AspPI* (aspartic proteinase inhibitor) gene expression showed a significantly higher induced response to herbivory (Figure 4B,E). While some studies report that salt‐stress by itself does not alter plant defense signalling (Thaler and Bostock 2004; Winter et al. 2012), others have reported salt‐stress‐induced accumulation of proteinase inhibitor II (*PIN2*) transcripts (Dombrowski 2003) as well as amplified plant defenses under combined salinity and insect herbivory (Forieri, Hildebrandt, and Rostás 2016). In our study, increased *PIN2* gene activity did not translate to higher levels of two downstream defense proteins: polyphenol oxidase (PPO) and trypsin proteinase inhibitor (TPI) in the combined salt and herbivory treatment (Ryan 1990; Tan et al. 2018; Thaler and Bostock 2004) (Figure S5 and S6, Notes S2).

Plant tissues with prior insect herbivory have elevated levels of plant defense proteins PPO and TPI, irrespective of salt treatment (Figures S5 and S6). This suggests that the observed impact of salt on caterpillar growth is likely not attributable to plant defense proteins, but due to salt itself. The additive negative effect observed when combining the salt treatment and prior herbivory is likely due to these factors operating through distinct mechanisms to impair caterpillar growth.

Under highly stressful salinity conditions, plants could be prioritizing nutrient homeostasis and growth over investing in defense metabolites (Abrol, Vyas, and Koul 2012). Furthermore, ionic toxicity to plant tissues could also inhibit enzymatic activity and reduce the accumulation of proteinase inhibitors (Deinlein et al. 2014; Joern, Provin, and Behmer 2012; Kaspari 2020; Marroquin, Holmes, and Salazar 2023). The influence of other defense metabolites such as alkaloids, phenolics, or flavonoids cannot be disregarded, although their specific impact was not measured in our study (Ballhorn and Elias 2014; Dong et al. 2020; Friedman 2002; Mahmoudi et al. 2010; Marroquin, Holmes, and Salazar 2023; Wahid and Ghazanfar 2006; Q. Wang et al. 2015). Lastly, it is crucial to recognize that the effects of salt stress on insect herbivores can vary depending on the insect species and the severity of the stress (Martínez et al. 2012). Contrary to previous studies, we find that salt alone does not induce the accumulation of proteinase inhibitors in tomatoes, and reduced caterpillar performance on salt‐treated plants is likely attributable to ionic toxicity rather than increased plant defense proteins.

### 4.4 Female *H. zea* Moths Prefer to Oviposit on Untreated Control Plants Over Salt‐Treated Plants

We see that female *H. zea* moths exhibit a clear preference for oviposition on no salt‐treated plants over salt‐treated plants (Figure 5A). This aligns with the “optimal oviposition theory” or “mother knows best hypothesis” which posits that female moths select oviposition sites that maximize the fitness and performance of their offspring (Courtney and Kibota 2017; Jaenike 1978; Valladares and Lawton 1991). Unlike our study, a study on monarchs showed no difference in oviposition between no salt and salt‐treated plants, highlighting the species‐specific impact of salt stress on insects (Hund et al. 2023; Mitchell et al. 2019).

For many noctuid lepidopterans, including *H. zea*, olfactory cues are crucial in host‐plant location and selection (Jost and Pitre 2002; McCallum et al. 2011; Rojas, Virgen, and Cruz‐López 2003). Volatile organic compounds (VOCs) emitted by plants can influence moth attraction and oviposition behavior (Bruce, Wadhams, and Woodcock 2005; De Moraes, Mescher, and Tumlinson 2001). VOC analysis showed decreased levels of β‐phellandrene, α‐pinene, β‐caryophyllene, α‐phellandrene, α‐humulene, and 2‐carene, in salt‐treated plants (Figure 5B–G). Our results differ from some other studies; for instance, the terpenes β‐phellandrene and 2‐carene were shown to be positively correlated with salt concentration (Tomescu et al. 2017). β‐phellandrene, α‐pinene, and other terpenoids have been implicated in attracting lepidopteran moths for oviposition (Agbessenou et al. 2022; D. Huang et al. 2020). β‐caryophyllene, as part of a volatile blend, has been shown to attract female *H. zea* moths in wind tunnels (Breeden et al. 1996; Douglass et al. 1993). Decreased constitutive emissions of β‐phellandrene, α‐pinene, and β‐caryophyllene from salt‐treated plants might have led to lower *H. zea* moth attraction, and subsequently, decreased oviposition. Apart from the emission of specific volatiles, ovipositing *H. zea* moths could be attracted to no salt‐treated plants based on differences to the overall blend (Figure 5I). Although the total volatiles emitted per biomass remained unchanged (Figure 5H), the total volatiles emitted by the plant could be a more ecologically relevant unit, with no salt‐treated plants simply “smelling” more than salt‐treated plants (Forieri, Hildebrandt, and Rostás 2016; Loreto and Delfine 2000; Teuber et al. 2008).

Our aim was to disentangle various mechanisms by which *H. zea* caterpillars could be affected by salinity stress. We determined that larval feeding preferences and growth were primarily driven by direct ionic toxicity, while salt‐induced ovipositional preferences of moths are likely driven by changes to plant volatile emissions. The observed feeding and oviposition preferences of *H. zea* larvae and moths may contribute to its success as a devastating crop pest. In natural settings, larval dispersal, mobility, and feeding choices could offset the negative effects of suboptimal oviposition choices (as we do report eggs laid on salt‐treated plants) (Hufnagel et al. 2017). There is also a need to understand how salt‐induced changes in plant‐insect interactions affect other trophic levels such as soil microbiomes and natural enemies of insect herbivores (Marroquin, Holmes, and Salazar 2023). These investigations will provide valuable insights into dynamics of insect pests in changing agricultural environments and may inform the development of novel pest management strategies.

## Conflicts of Interest

The authors declare no conflicts of interest.

## Supporting information

Supporting information.

## Data Availability

The data that support the findings of this study are available from the corresponding author upon reasonable request.
