# Supplementary material for: Tomato Defenses Under Stress: The Impact of Salinity on Direct Defenses Against Insect Herbivores
Source: Plant Cell Environ. 2025 Jan 13;48(5):3647–59. doi: 10.1111/pce.15353 (PMC11963492; doi:10.1111/pce.15353)
Supplement: Supplementary file 1 — Supporting information. [file PCE-48-3647-s001.docx]

**Supplementary Information**

The following Supplementary Materials are available for this article:


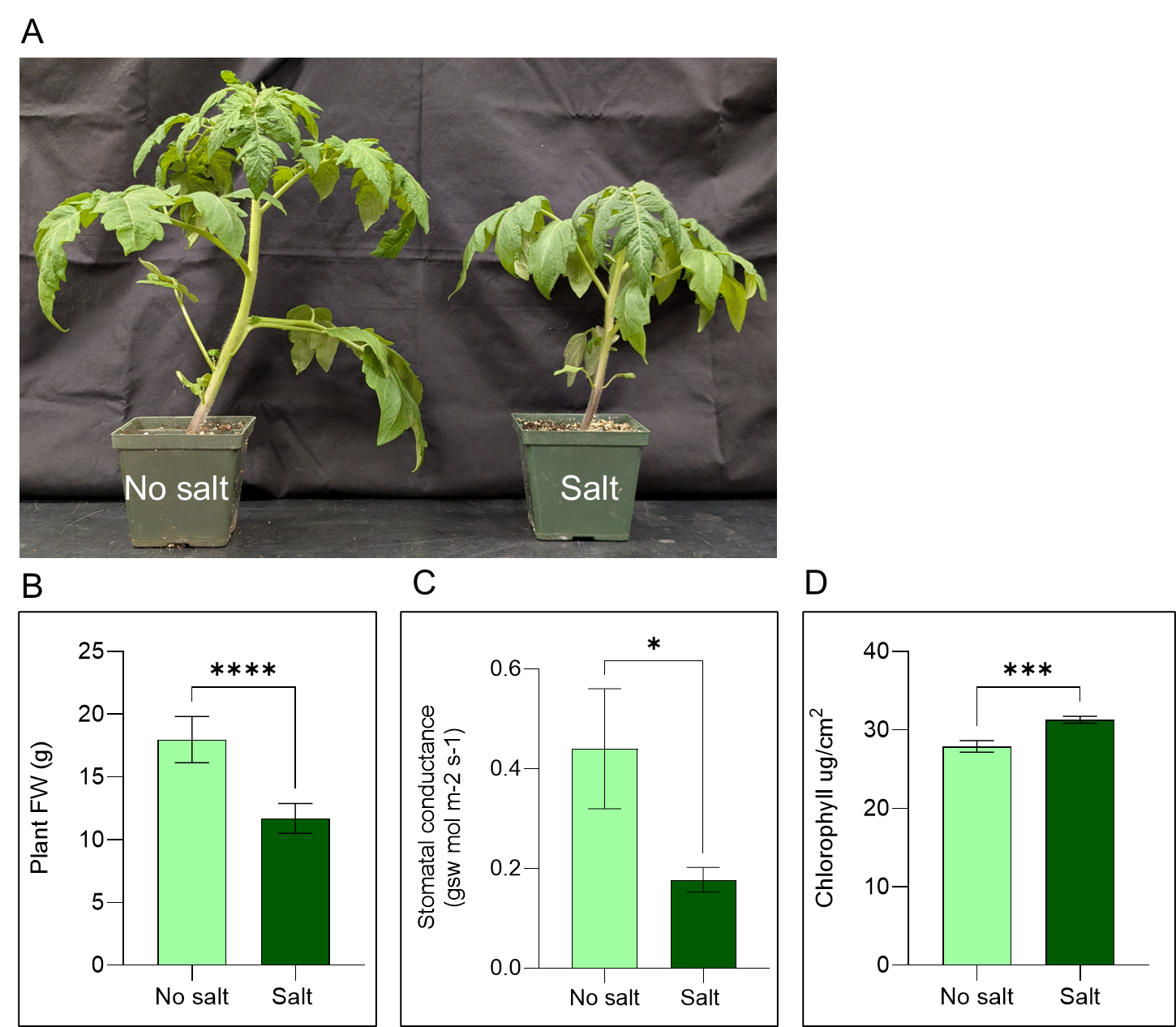


**Supplementary Figure S1:** A. Phenotypes of tomato plants growing under No salt and Salt treatments. B. Salt treatment significantly reduced plant fresh weight from 17.98 ± 1.852 g in no salt-treated plants to 11.70 ± 1.189 g in salt-treated plants (Student’s T-test, n = 10, t = 6.895, df = 9, *P* < 0.001). C. Tomato plants showed significantly lower stomatal conductance post salt treatment (Student’s T-test, n = 18, t = 2.258, df = 32, *P* < 0.05). D. Plants appeared significantly darker in the salt treatment due to significantly higher chlorophyll content (Student’s T-test, n = 36, t = 3.947, df = 70, *P* = 0.0002).

**
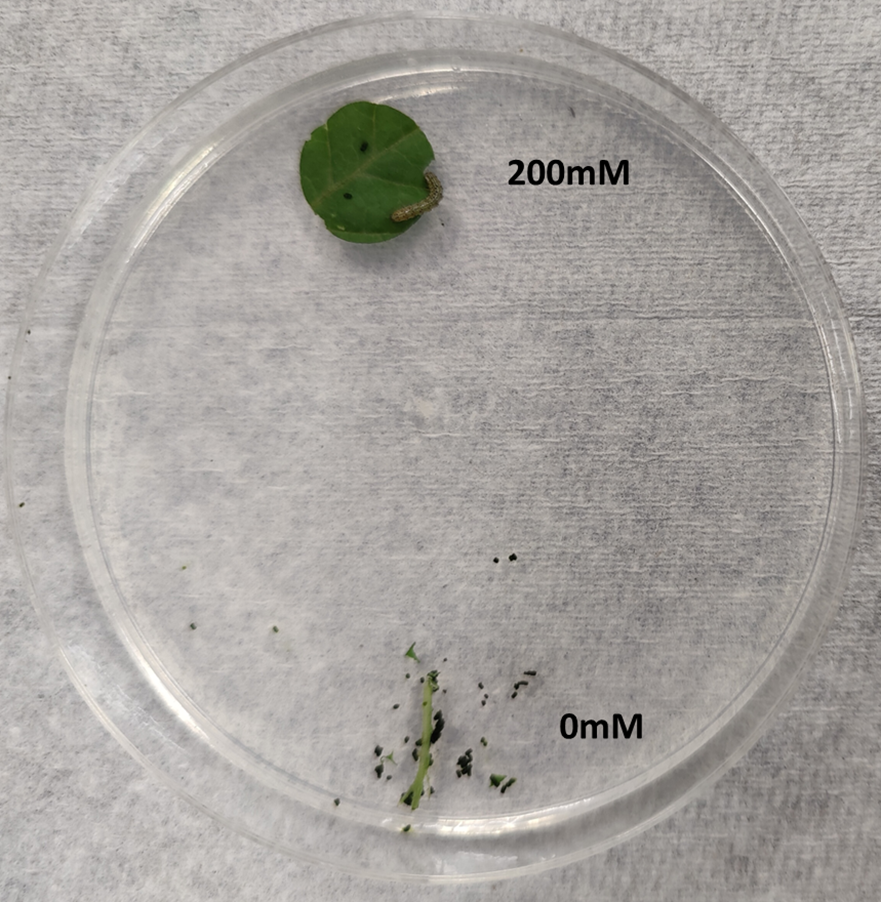
**

**Supplementary Figure S2:** Visual representation of first finish in a Petri dish-based two-choice test. 0 mM leaf has been “finished first”.

**
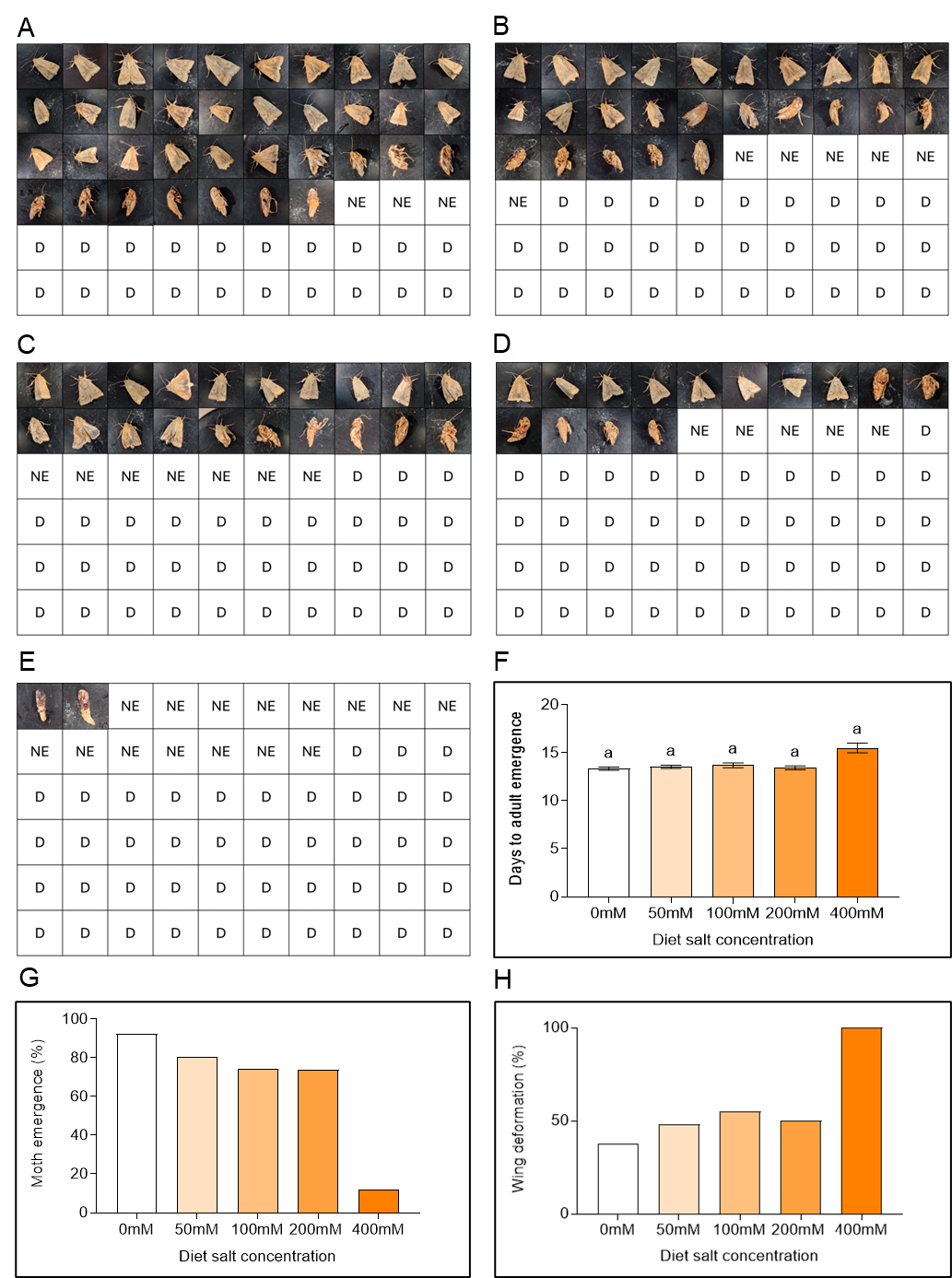
**

**Supplementary Figure S3:** Fate of *H. zea* larvae placed on artificial diets differing in salt concentrations (A: 0 mM; B: 50 mM; C: 100 mM; D: 200 mM; E: 400 mM). Each block corresponds to a single insect, with a total of 60 insects per treatment. NE (not emerged) represents adult moths that failed to emerge from their pupae. D (dead) represents insects that died before they reached pupation. F. Average number of days spent as a pupa before emerging as an adult remained unchanged between treatments (Bars represent mean ± SEM, Kruskal-Wallis statistic = 7.347, df = 5, *P* = 0.1187). G. Percentage moth emergence – The % of adult *H. zea* moths emerging from pupae decreased with increasing salt concentration in insect diet (92.5% in 0 mM, 80.64% in 50 mM, 74.07% in 100mM, 73.68% in 200 mM and 11.76% in 400mM). H. Percentage wing deformation - The percentage of moths emerging with deformed wings and improper development increased with increasing salt concentration in insect diet (37.83% in 0 mM, 48% in 50 mM, 55 % in 100 mM, 50% in 200 mM, and 100% in 400 mM).


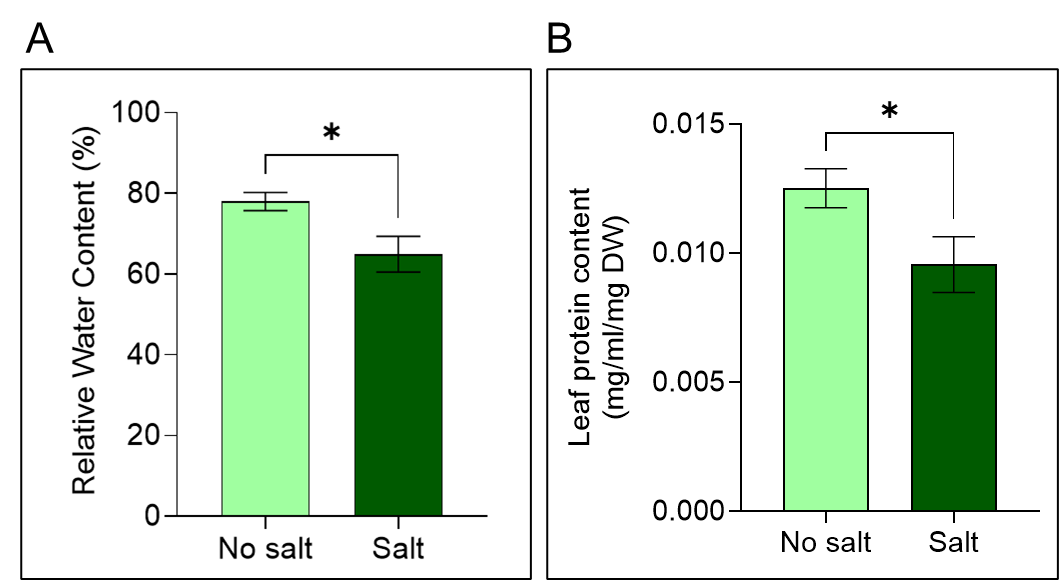


**Supplementary Figure S4: Quantifying plant nutritional quality.** Salt application significantly decreased A. leaf relative water content (RWC) from 78 ± 2.25% in no salt-treated plant leaves to 64.92 ± 4.45% in salt-treated plant leaves and B. leaf total protein content from 0.012 ± 0.0007 mg/ ml/ mg dry weight in no salt-treated plant leaves to 0.0095 ± 0.0010 mg/ ml/ mg dry weight in salt-treated plant leaves. Bars represent mean (± SEM) (A. Student’s T-test, t = 2.621, df = 16, *P* < 0.05; B. Student’s T-test, t = 2.245, df =12, *P* < 0.05). See **Supplementary Notes S1** for experiment methods.

**
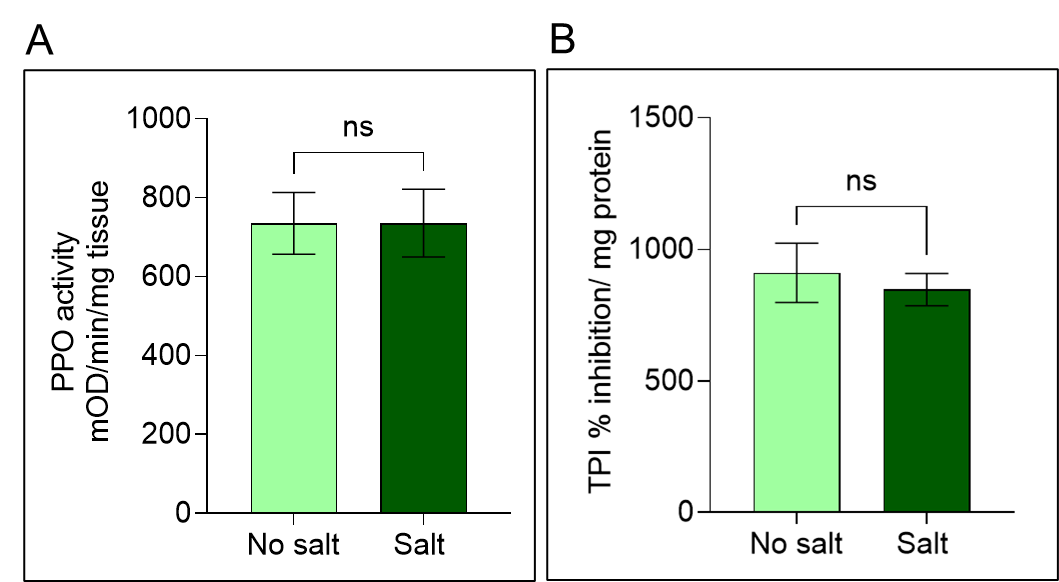
**

**Supplementary Figure S5: Plant defense proteins.** Salt application alone does not influence PPO and TPI levels. A. PPO and B. TPI levels did not significantly differ between no salt-treated and salt-treated leaves three days post salt application. Bars represent mean (± SEM) (n = 9, Student’s T-test, all *P* > 0.05). See **Supplementary Notes S2** for experiment methods.


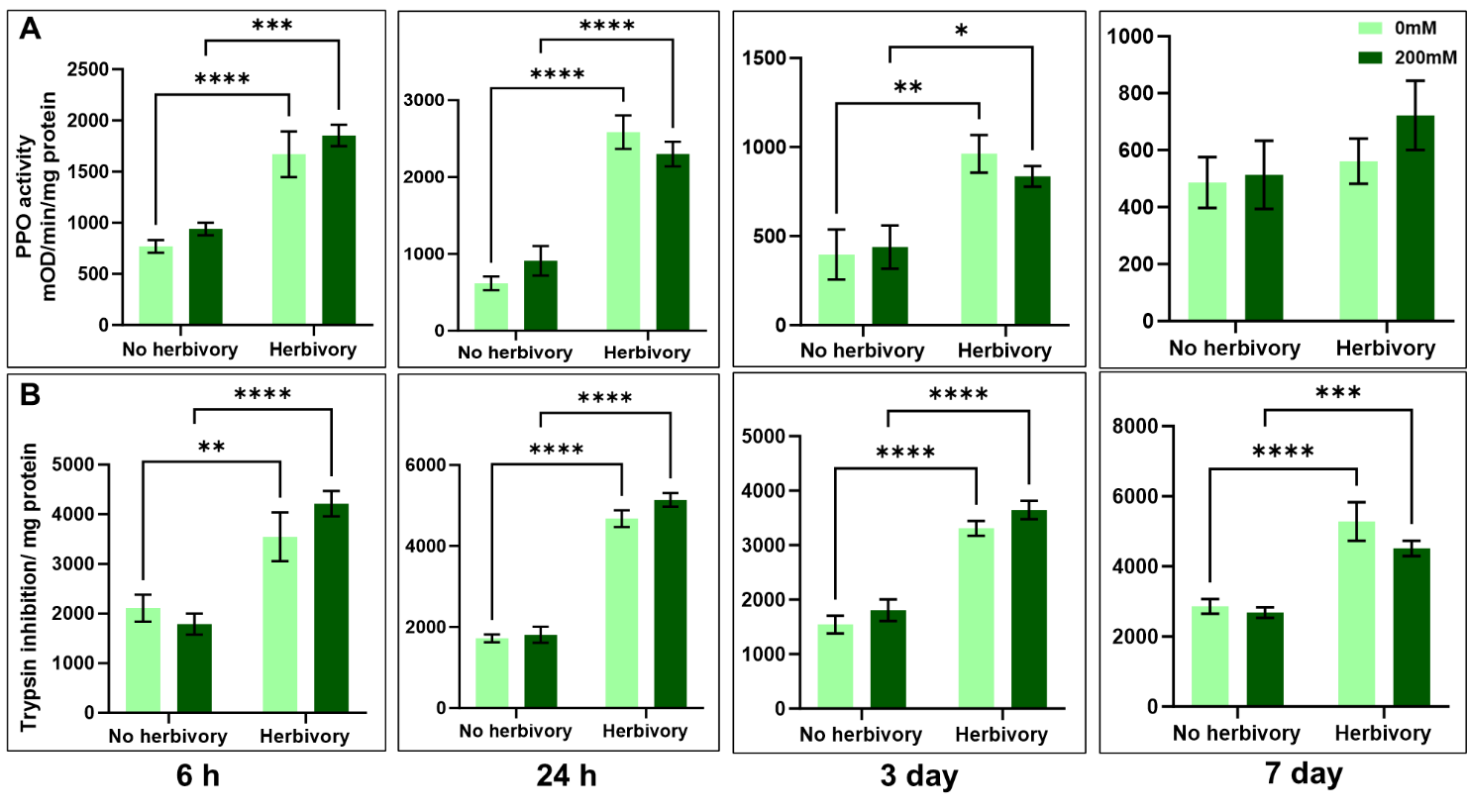


**Supplementary Figure S6: Plant defense proteins.** Salt addition over short (6 h, 24 h) and long (3 days, 7 days) durations does not prime plants for a higher induced A. PPO and B. TPI response to insect herbivory. There was no significant interaction between salt and herbivory on PPO and TPI levels at all time intervals (all *P* > 0.05). Furthermore, salt application had no effect on PPO and TPI levels, regardless of insect herbivory (all *P* > 0.05). Herbivory, however, led to a significant increase in the PPO activity and TPI levels, regardless of salt application (all *P* < 0.05). There were no significant increases in PPO levels after herbivory at 7 days of salt treatment (*P* > 0.05). Bars represent mean (± SEM) (n = 8-11, mixed-effects analysis, * *P* < 0.05, ** *P* < 0.01, *** *P* < 0.001, **** *P* < 0.0001).

**Supplementary Table S1:** **Quantifying plant nutritional quality.** Element levels in *Solanum lycopersicum* cv. Better Boy leaves exposed to 0 mM and 200 mM NaCl. Values represent mean ± SEM (n = 6).

| **Element** | **No salt (0mM)** | **Salt (200 mM)** | **t, df** | ***P* value** |
| --- | --- | --- | --- | --- |
| P (%) | 0.7950 ± 0.0352 | 0.8483 ± 0.0181 | t = 1.344, df = 10 | *P* = 0.2086 |
| K (%) | 4.188 ± 0.2391 | 4.708 ± 0.1259 | t = 1.925, df =10 | *P* = 0.0832 |
| Ca (%) | 1.618 ± 0.1011 | 1.997 ± 0.0653 | t = 3.143, df =10 | *P* = 0.0105 |
| Mg (%) | 0.34 ± 0.01 | 0.4167 ± 0.0156 | t = 4.131, df =10 | *P* = 0.0020 |
| S (%) | 0.6217 ± 0.0107 | 0.4983 ± 0.0122 | t = 7.568, df =10 | *P* < 0.0001 |
| Mn (mg/kg) | 61 ± 5.508 | 111.3 ± 6.020 | t = 6.169, df =10 | *P =* 0.0001 |
| Zn (mg/kg) | 77.83 ± 2.072 | 103.2 ± 4.445 | t = 5.165, df =10 | *P =* 0.0004 |
| Cu (mg/kg) | 14.50 ± 0.3416 | 12.50 ± 0.6191 | t = 2.828, df =10 | *P* = 0.0179 |
| B (mg/kg) | 41.67 ± 0.7149 | 32.50 ± 1.522 | t = 5.451, df =10 | *P =* 0.0003 |
| Al (mg/kg) | 5 ± 0.4472 | 4.8 ± 0.2 | t = 0.3800, df =10 | *P* = 0.7128 |
| Fe (mg/kg) | 88.33 ± 1.116 | 86.83 ± 2.197 | t = 0.6087, df =10 | *P* = 0.5563 |
| Na (mg/kg) | 786.8 ± 63.83 | 10497 ± 374.8 | t = 25.54, df = 10 | *P* < 0.0001 |

**Supplementary Table S2:** Salt treatment-based tomato volatile changes. Amounts of VOCs (ng/gm fresh weight ± SEM) detected in GC-MS analysis (n = 10, Student’s T-test in no salt and salt-treated plants, * *P* < 0.05, ** *P* < 0.01).

| **Compound** | **Classification** | **No salt (ng/gm FW)** | **Salt (ng/gm FW)** | ***P* value** |
| --- | --- | --- | --- | --- |
| (Z)-3-hexenol | GLV^a^ | 0.39 ± 0.08 | 0.68 ± 0.16 | 0.1286 |
| β-phellandrene | Terpene | 132.9 ± 13.14 | 72.87 ± 10.84 | 0.0024** |
| α-pinene | Terpene | 5.35 ± 0.52 | 3.57 ± 0.45 | 0.0197* |
| β-caryophyllene | Terpene | 2.66 ± 0.79 | 0.56 ± 0.09 | 0.0171* |
| α-phellandrene | Terpene | 10.51 ± 1.51 | 4.00 ± 0.50 | 0.0007*** |
| α-humulene | Terpene | 0.67 ± 0.13 | 0.28 ± 0.05 | 0.0152* |
| 2-carene | Terpene | 36.19 ± 4.26 | 19.94 ± 2.91 | 0.0055** |
| Citronellene | Terpene | 8.25 ± 2.08 | 12.50 ± 2.95 | 0.2567 |
| Cumene | Aromatic | 12.42 ± 2.72 | 21.02 ± 5.18 | 0.1593 |
| Mesitylene | Aromatic | 65.76 ± 13.55 | 98.73 ± 22.64 | 0.2274 |
| Hemimellitene | Aromatic | 29.90 ± 6.25 | 43.88 ± 10.33 | 0.2622 |
| 3,7,7-Trimethylcyclohepta-1,3,5-triene | Aromatic | 13.90 ± 1.22 | 13.26 ± 2.21 | 0.8033 |
| Sulcatone | Ketone | 11.62 ± 3.03 | 15.61 ± 2.94 | 0.3567 |
| β-Myrcene | Terpene | 5.93 ± 0.78 | 5.00 ± 0.91 | 0.4529 |
| α-Terpinene | Terpene | 5.44 ± 0.48 | 3.79 ± 0.88 | 0.1220 |
| Indane | Hydrocarbon | 5.83 ± 1.41 | 6.93 ± 1.84 | 0.6424 |
| β-Ocimene | Terpene | 4.12 ± 0.53 | 4.76 ± 0.89 | 0.5414 |
| Terpinolene | Terpene | 2.99 ± 0.29 | 3.10 ± 0.72 | 0.8891 |
| 2-methyl-trans-Decalin | Hydrocarbon | 3.04 ± 0.51 | 3.88 ± 0.73 | 0.3625 |
| Pentylcyclohexane | Alcohol | 0.92 ± 0.17 | 1.34 ± 0.28 | 0.2254 |
| 3-Hexenyl butyrate | Ester | 0.83 ± 0.08 | 0.96 ± 0.13 | 0.4212 |
| Cryptone | Cyclohexenone | 1.33 ± 0.29 | 2.54 ± 0.89 | 0.2152 |
| Methyl salicylate | Ester | 2.75 ± 0.82 | 3.41 ± 0.59 | 0.5187 |
| Decanal | Aldehyde | 4.66 ± 1.17 | 6.93 ± 1.13 | 0.1793 |
| 1,4-Diacetylbenzene | Aromatic | 3.91 ± 0.92 | 4.82 ± 1.03 | 0.5205 |
| Geranyl acetone | Terpenoid | 1.99 ± 0.49 | 2.19 ± 0.49 | 0.7793 |
| 3E,7E)-4,8,12-Trimethyltrideca-1,3,7,11-tetraene (TMTT) | GLV | 30.92 ± 13.34 | 30.35 ± 13.62 | 0.9764 |

^a^ GLV – Green Leaf Volatiles

**Supplementary Table S3:** qRT-PCR primer pairs used for tomato gene expression.

| **Gene name** | **Description** | **Forward Primer** | **Reverse Primer** | **Accession no.** | **References** |
| --- | --- | --- | --- | --- | --- |
| *PIN2* | Wound-induced  proteinase inhibitor 2 | GGATTTAGCGGACTTCCTTCTG | ATGCCAAGGCTTGTACTAGAGAATG | K03291 | (Tan *et al.*, 2018) |
| *TD2* | Threonine deaminase 2 | CCCTGGGAGGTGATGTAGTTCT | TCGAATGGTGGGATGTATTTGAG | M61915 | (Tan *et al.*, 2018) |
| *AspPI* | Aspartic proteinase  inhibitor | AGCCAGTCCTTGACACAAGTGGTA | AGGTACACATCACCACCTAACGCA | SGN-U143342^a^ | (Tan *et al.*, 2018) |
| *PPOB* | Polyphenol oxidase B | TTCGCGAGTGGGAATACCTCGTTT | AGTCAGGGACTGTTTGGACACGAA | Z12834 | (Tan *et al.*, 2019) |
| *CysPI* | Cysteine proteinase inhibitor | GGTGAAGGAATGGGAGGACT TCAA | GGAGGTTTGGGAATGGAACA TTGG | AF198390 | (Tan *et al.*, 2019) |
| *UBI* | Ubiquitin | GCCAAGATCCAGGACAAGGA | GCTGCTTTCCGGCGAAA | X58253 | (Tan *et al.*, 2019) |

^a^ Solanaceous Gene Network (SGN) Unigene id

**Supplementary Table S4:** Mixed effects model results for gene expression.

| **Gene** | **Herbivory** | **Salt** | **Herbivory X Salt** |
| --- | --- | --- | --- |
| *PIN2* | F _(1, 8)_ = 19.20, *P* = 0.0023 | F _(1, 10)_ = 1.885, *P* = 0.1997 | F _(1, 8)_ = 5.017, *P* = 0.0554 |
| *TD2* | F _(1, 8)_ = 30.69, *P* = 0.0005 | F _(1, 10)_ = 0.9825, *P* = 0.345 | F _(1, 8)_ = 2.812, *P* = 0.1321 |
| *PPOB* | F _(1, 5)_ = 8.79, *P* = 0.0313 | F _(1, 10)_ = 6.063, *P* = 0.0335 | F _(1, 5)_ = 0.8776, *P* = 0.3919 |
| *AspPI* | F _(1, 18)_ = 4.835, *P* = 0.0412 | F _(1, 18)_ = 0.4075, *P* = 0.5313 | F _(1, 18)_ = 3.03, *P* = 0.0988 |
| *CysPI* | F _(1, 7)_ = 7.193, *P* = 0.0314 | F _(1, 10)_ = 0.0253, *P* = 0.8768 | F _(1, 7)_ = 0.0026, *P* = 0.9605 |

**Supplementary Notes S1: Quantifying plant relative water content (RWC) and total protein content**

Leaf relative water contents (RWC) were estimated using the method as described by Mullan & Pietragalla, 2012) (n = 9). Leaf total protein content was estimated using a Bradford protein assay using a bovine serum albumin standard curve (Bradford, 1976) (n = 10).

**Supplementary Notes S2: Plant defense proteins**

PPO and TPI activity levels were measured after salt addition alone after three days (**Supplementary Figure S5**) (n = 9). PPO and TPI activity levels were measured after short term (6 hours, 24 hours) and long term (3 days, 7 days) salt treatment followed by insect herbivory (**Supplementary Figure S6**). A two-factorial assay with salt (0 mM and 200 mM) and herbivory (no herbivory and herbivory) was conducted. A single, one-day starved 5^th^ instar *H. zea* caterpillar was allowed to feed inside a clip cage (3.15 cm^2 ­­^ leaf area) on the fourth fully expanded leaf of the plant as part of the herbivory treatment. Empty clip cages were placed on plants in the no herbivory treatment. Forty-eight hours after tomato plants were subjected to their respective treatments, 50 mg of leaf tissue was collected in liquid nitrogen and stored at -80°C until further analysis. PPO and TPI levels were analysed using a spectrophotometric method (Acevedo *et al.*, 2017). Leaf total protein content was estimated using a Bradford protein assay using a bovine serum albumin standard curve (Bradford, 1976) (n = 8 – 11).

**Supplementary Notes S3: Quantifying caterpillar frass content levels**

No salt and salt-treated plant tissue was fed to *H. zea* larvae. Frass from no salt and salt-treated-plant fed larvae was collected and sent for ion content analysis to the Penn State Analytical Laboratory ((Huang and Schulte, 1985). Frass from 40 caterpillars of each treatment was pooled. Average Na^+^ levels were 70 mg/kg in no salt frass samples and was 1855 mg/kg in salt frass samples.

**References**

Acevedo, F. E., Peiffer, M., Tan, C.-W., Stanley, B. A., Stanley, A., Wang, J., Jones, A. G., Hoover, K., Rosa, C., Luthe, D., & Felton, G. (2017). Fall Armyworm-Associated Gut Bacteria Modulate Plant Defense Responses. *Molecular Plant-Microbe Interactions®*, *30*(2), 127–137. https://doi.org/10.1094/MPMI-11-16-0240-R

Bradford, M. M. (1976). A rapid and sensitive method for the quantitation of microgram quantities of protein utilizing the principle of protein-dye binding. *Analytical Biochemistry*, *72*(1–2), 248–254.

Huang, C. L., & Schulte, E. (1985). Digestion of plant tissue for analysis by ICP emission spectroscopy. *Communications in Soil Science and Plant Analysis*, *16*(9), 943–958.

Mullan, D., & Pietragalla, J. (2012). Leaf relative water content. *Physiological Breeding II: A Field Guide to Wheat Phenotyping*, 25–27.

Tan, C.-W., Peiffer, M., Hoover, K., Rosa, C., Acevedo, F. E., & Felton, G. W. (2018). Symbiotic polydnavirus of a parasite manipulates caterpillar and plant immunity. *Proceedings of the National Academy of Sciences*, *115*(20), 5199–5204. https://doi.org/10.1073/pnas.1717934115

Tan, C.-W., Peiffer, M., Hoover, K., Rosa, C., & Felton, G. W. (2019). Parasitic wasp mediates plant perception of insect herbivores. *Journal of Chemical Ecology*, *45*, 972–981.
